# Supplementary material for: Dietary Fiber Intake Influences Changes in Ankylosing Spondylitis Disease Status
Source: J Clin Med. 2023 Feb 17;12(4):1621. doi: 10.3390/jcm12041621 (PMC9960917; doi:10.3390/jcm12041621)
Supplement: Supplementary file 1 [file jcm-12-01621-s001.zip › FFQ Questionnaire.pdf]

Semi-quantitative Food Frequency Questionnaire (AS-FMT)

1. Name [fill in the blank] *

____

2. gender [Single-choice] *

○Men

○Girl

3. How often do you eat the following rice and pasta? How many grams do you eat each time? (Please estimate based on the picture prompt and answer in grams in the option description.) [Matrix Single Choice] *

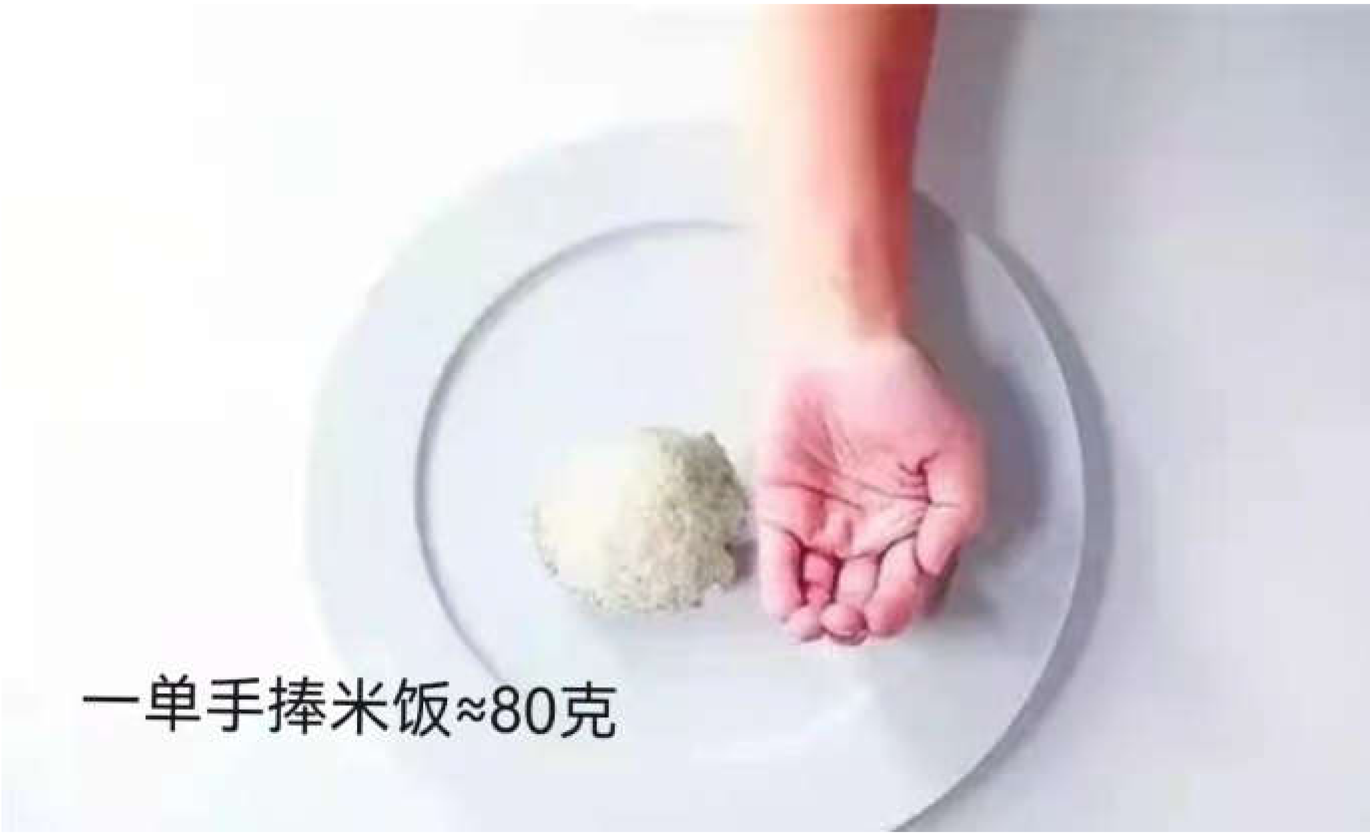

	Basic	1 time	2-3	1 time	2	3-4	5-6	1 time	≥2

	ally don't eat	per month	times per month	per week	times a week	times per week	times per week	per day	times per day
Rice	○	○	○	○	○	○	○	○	○
Congee	○	○	○	○	○	○	○	○	○
Noodles	○	○	○	○	○	○	○	○	○
Dumplings / Buns	○	○	○	○	○	○	○	○	○
Steamed buns/flower rolls/cooked buns	○	○	○	○	○	○	○	○	○
Bread	○	○	○	○	○	○	○	○	○

4. How often do you eat the following potatoes? How many grams do you eat each time? (Please estimate based on the picture prompt and answer in grams in the option description.) [Matrix Single Choice] *

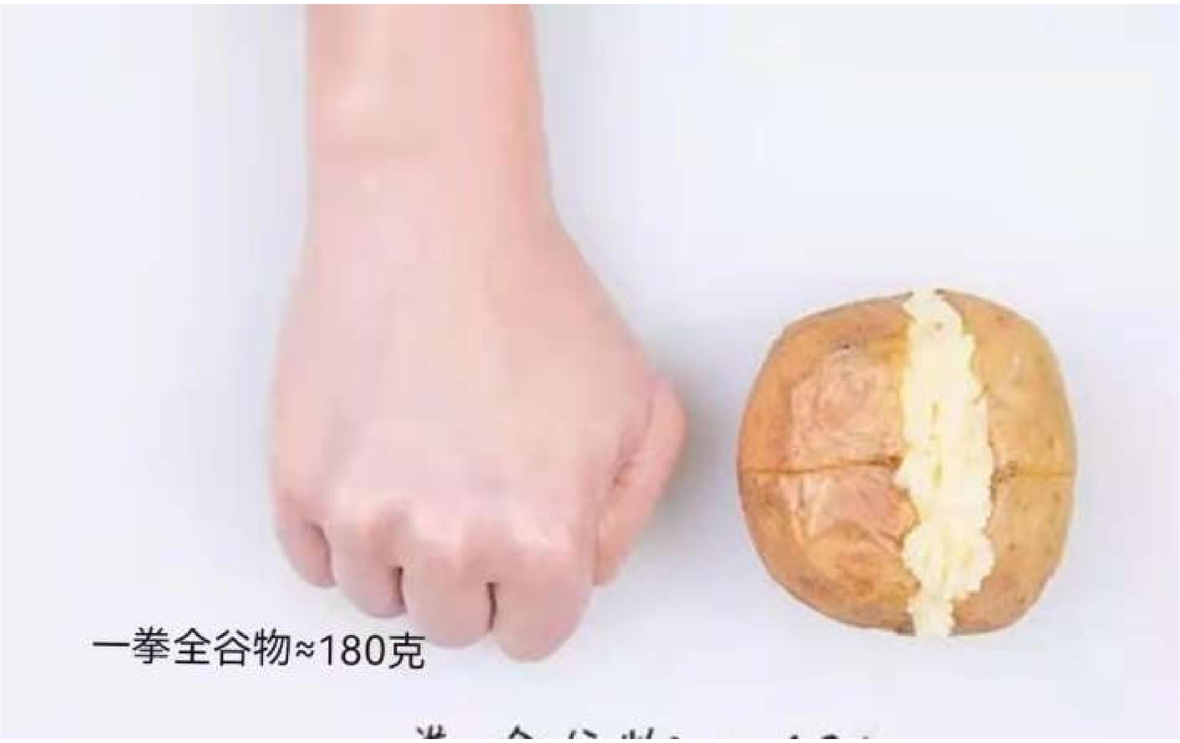

	Basically don't eat	1 time per month	2-3 times per month	1 time per week	2 times a week	3-4 times per week	5-6 times per week	1 time per day	≥2 times per day
Potatoes	○	○	○	○	○	○	○	○	○
Sweet Potato	○	○	○	○	○	○	○	○	○
Taro	○	○	○	○	○	○	○	○	○

5. How often do you eat the following mixed/coarse grains? How many grams do you eat each time? (Please estimate based on the picture prompt and answer in grams in the option description.) [Matrix Single Choice] *

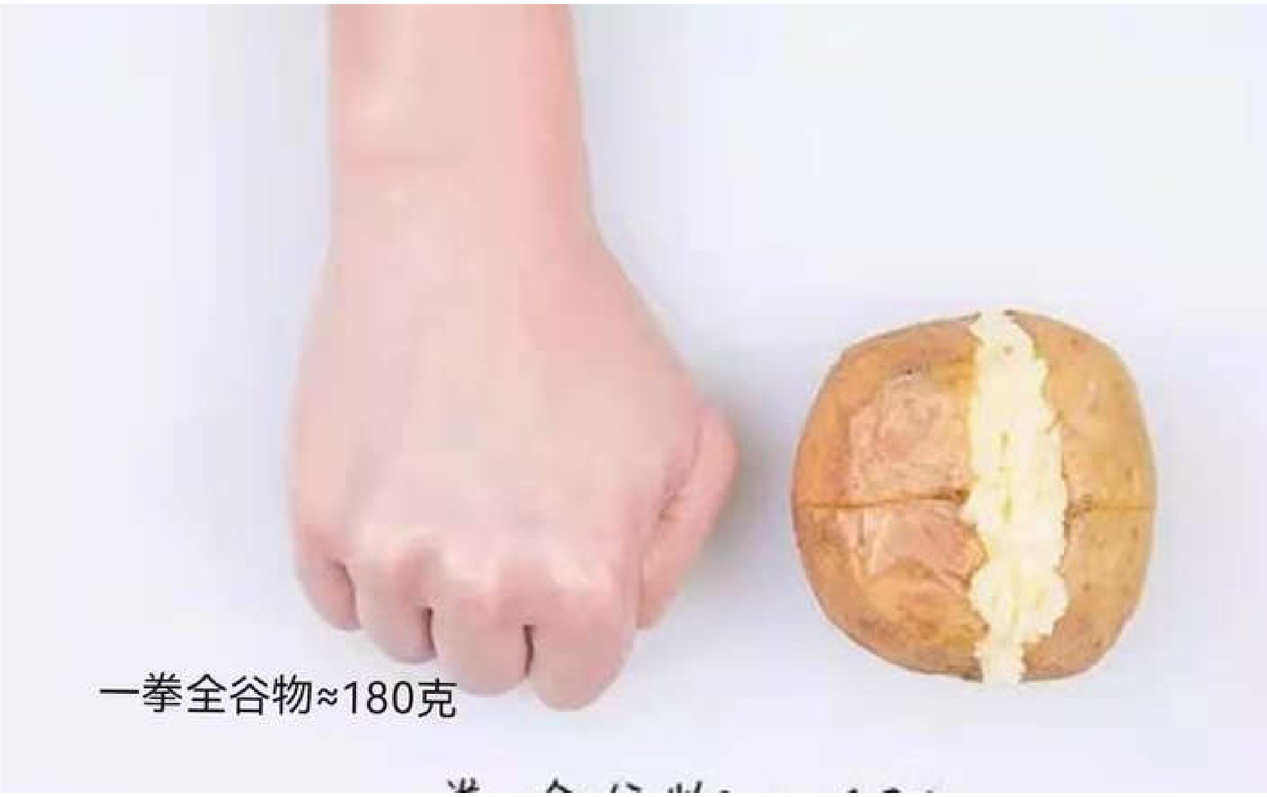

	Basically don't eat	1 time per month	2-3 times per month	1 time per week	2 times a week	3-4 times per week	5-6 times per week	1 time per day	≥2 times per day
Xiaomi	○	○	○	○	○	○	○	○	○
Oatmeal	○	○	○	○	○	○	○	○	○
Corn	○	○	○	○	○	○	○	○	○

6. How often do you eat the following mixed legumes? How many grams do you eat each time? (Please estimate based on the picture prompt and answer in grams in the option description.) [Matrix Single Choice] *

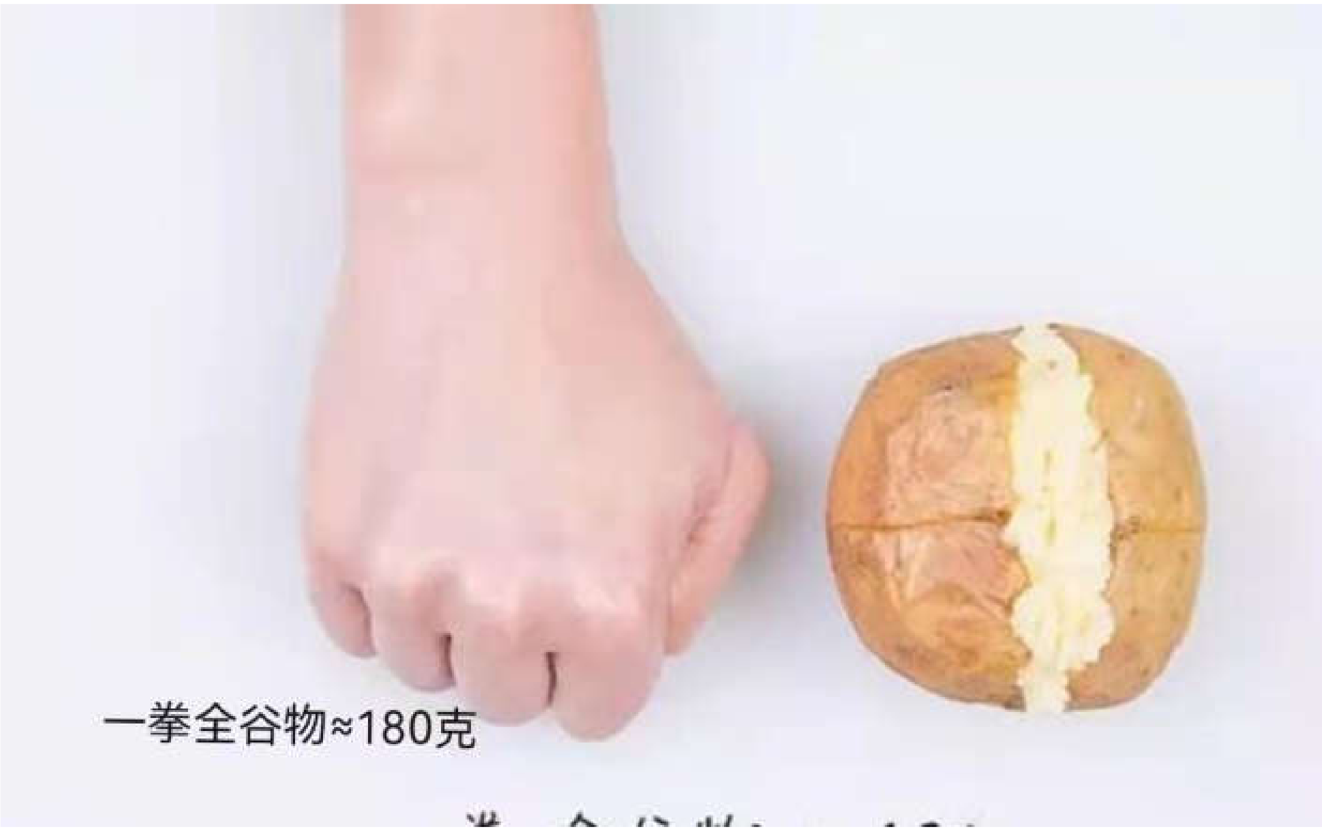

	Basically don't eat	1 time per month	2-3 times per month	1 time per week	2 times a week	3-4 times per week	5-6 times per week	1 time per day	≥2 times per day
Kidney beans	○	○	○	○	○	○	○	○	○
Chickpeas	○	○	○	○	○	○	○	○	○
Mung beans	○	○	○	○	○	○	○	○	○
Fava beans	○	○	○	○	○	○	○	○	○

7. How often do you eat the following tofu and its products? How many grams do you eat each time? (Please estimate based on the picture prompt and answer in the option description in grams.) [Matrix Single-Choice Question] *

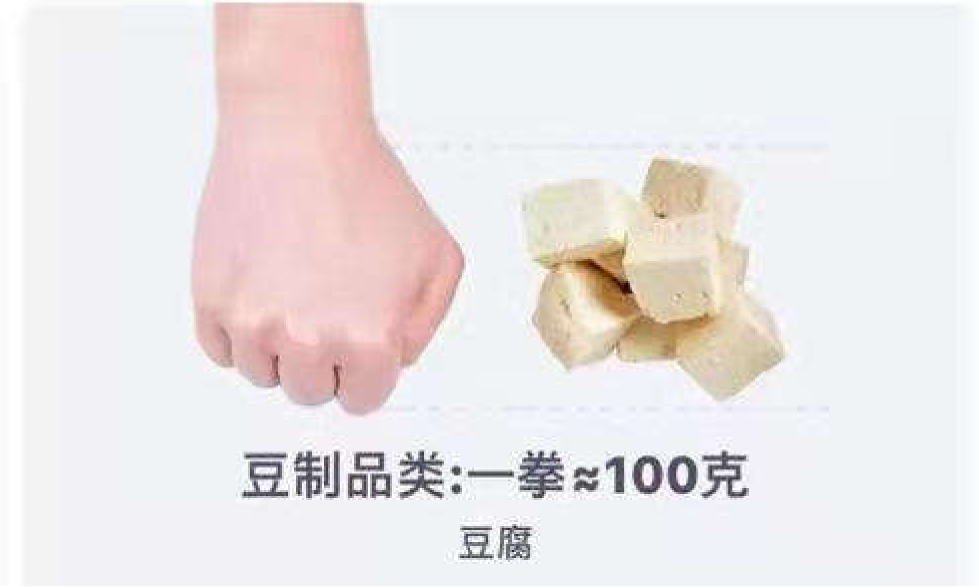

	Basically don't eat	1 time per month	2-3 times per month	1 time per week	2 times a week	3-4 times per week	5-6 times per week	1 time per day	≥2 times per day
Tofu	○	○	○	○	○	○	○	○	○
Dried bean curd/skin	○	○	○	○	○	○	○	○	○
Roti	○	○	○	○	○	○	○	○	○
Soy milk	○	○	○	○	○	○	○	○	○

8. How often do you eat the following fresh bean vegetables? How many grams do you eat each time? (Please estimate based on the picture prompt and answer in grams in the option description.) [Matrix Single Choice] *

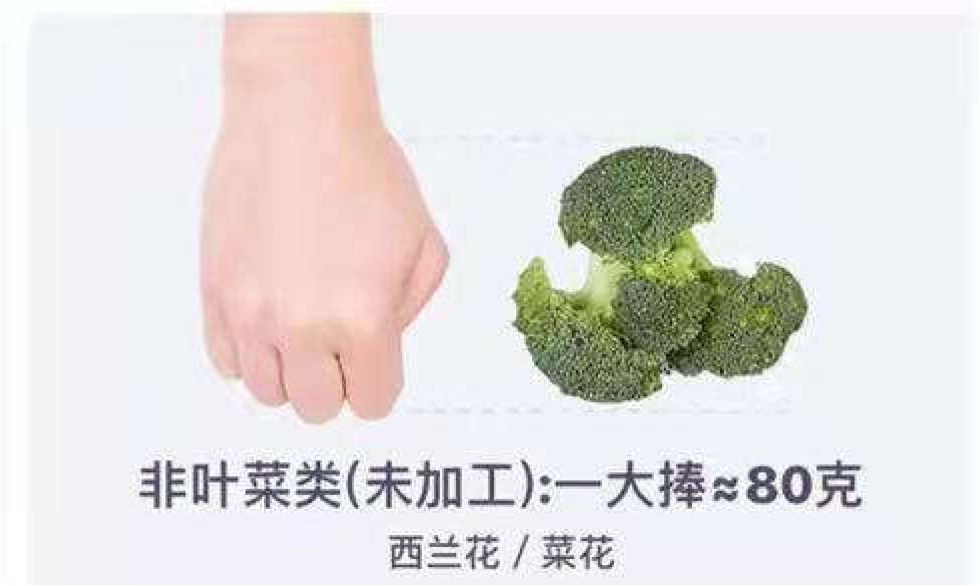

	Basically don't eat	1 time per month	2-3 times per month	1 time per week	2 times a week	3-4 times per week	5-6 times per week	1 time per day	≥2 times per day
Peas	○	○	○	○	○	○	○	○	○
Cowpea (bean curd)	○	○	○	○	○	○	○	○	○
Lentils	○	○	○	○	○	○	○	○	○
Quadrangular beans	○	○	○	○	○	○	○	○	○
Dutch beans	○	○	○	○	○	○	○	○	○
bean seedling	○	○	○	○	○	○	○	○	○

9. How often do you eat the following green leafy vegetables? How many grams do you eat each time? (Please estimate based on the picture prompt and answer in grams in the option description.) [Matrix Single Choice] *

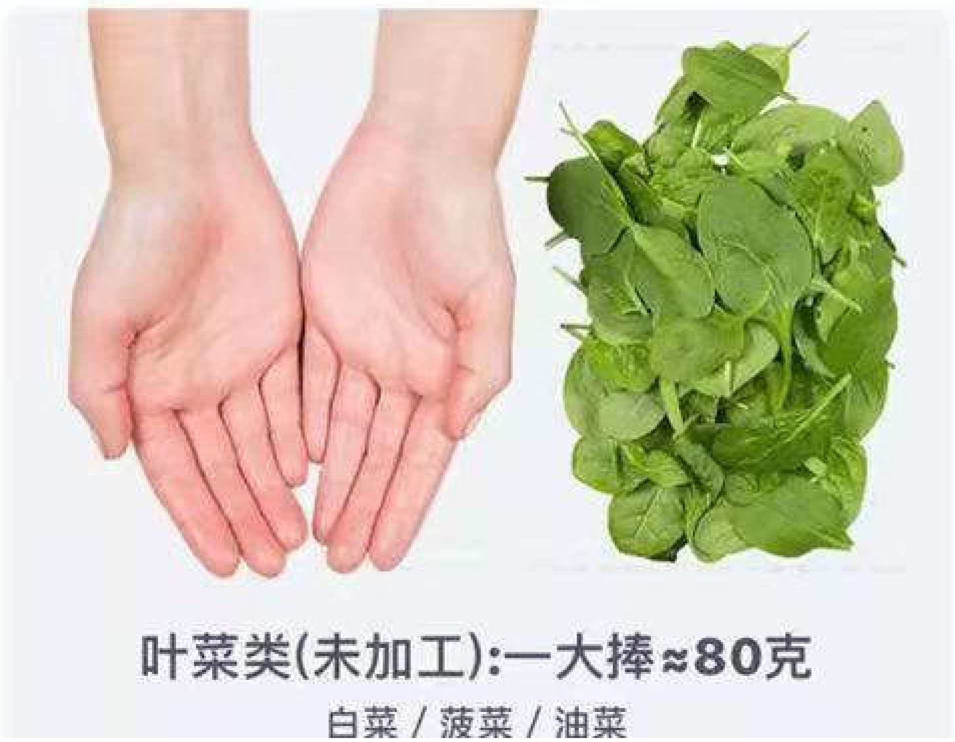

	Basically don't eat	1 time per month	2-3 times per month	1 time per week	2 times a week	3-4 times per week	5-6 times per week	1 time per day	≥2 times per day
Oilseed rape	○	○	○	○	○	○	○	○	○
Spinach	○	○	○	○	○	○	○	○	○
Lettuce	○	○	○	○	○	○	○	○	○
Cabbage	○	○	○	○	○	○	○	○	○
Nectarine	○	○	○	○	○	○	○	○	○
Chinese cabbage	○	○	○	○	○	○	○	○	○
Chinese cabbage	○	○	○	○	○	○	○	○	○

(Chinese cabbage)									
Chrysanthemum coronarium	○	○	○	○	○	○	○	○	○
Cilantro	○	○	○	○	○	○	○	○	○
Celery	○	○	○	○	○	○	○	○	○
Kale (Round cabbage)	○	○	○	○	○	○	○	○	○

10. How often do you eat the following squash vegetables? How many grams do you eat each time? (Please estimate based on the picture prompt and answer in grams in the option description.) [Matrix Single Choice] *

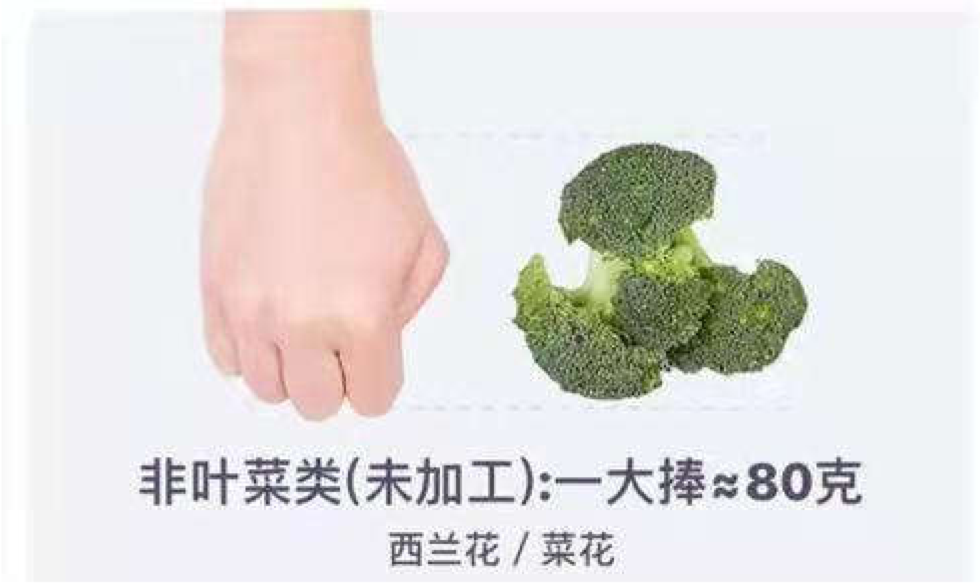

	Basically don't eat	1 time per month	2-3 times per month	1 time per week	2 times a week	3-4 times per week	5-6 times per week	1 time per day	≥2 times per day

Winter Melon	○	○	○	○	○	○	○	○	○
Cucumber	○	○	○	○	○	○	○	○	○
Loofah	○	○	○	○	○	○	○	○	○
Pumpkin	○	○	○	○	○	○	○	○	○
Zucchini	○	○	○	○	○	○	○	○	○

11. How often do you eat the following cauliflower vegetables? How many grams do you eat each time? (Please estimate based on the picture prompt and answer in grams in the option description.) [Matrix Single Choice] *

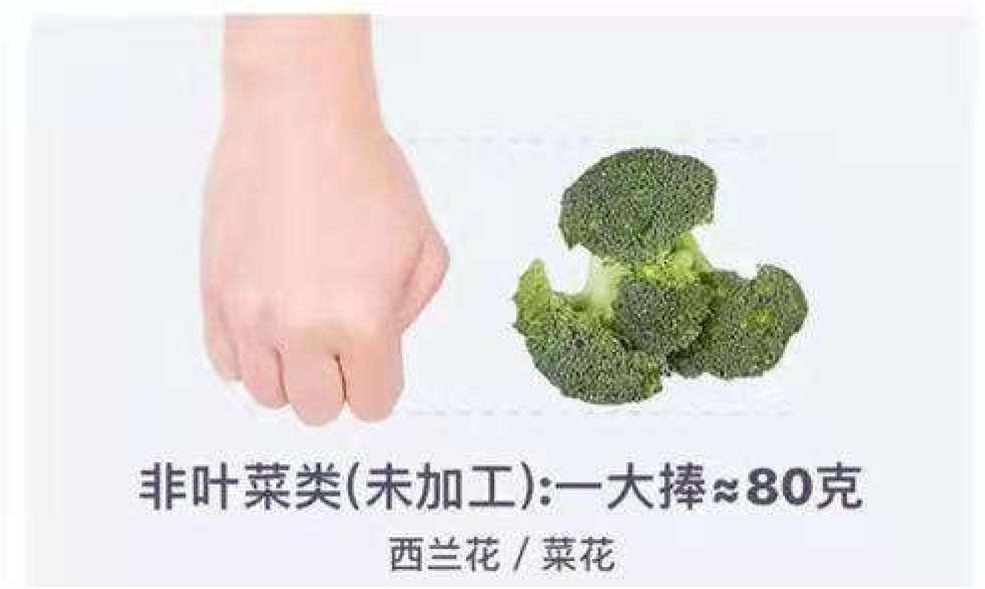

	Basically don't eat	1 time per month	2-3 times per month	1 time per week	2 times a week	3-4 times per week	5-6 times per week	1 time per day	≥2 times per day
Cauliflower	○	○	○	○	○	○	○	○	○
Broccoli	○	○	○	○	○	○	○	○	○

12. How often do you eat the following onion and garlic vegetables? How many grams do you eat each time? (Please estimate based on the picture prompt and answer in grams in the option description.) [Matrix Single Choice] *

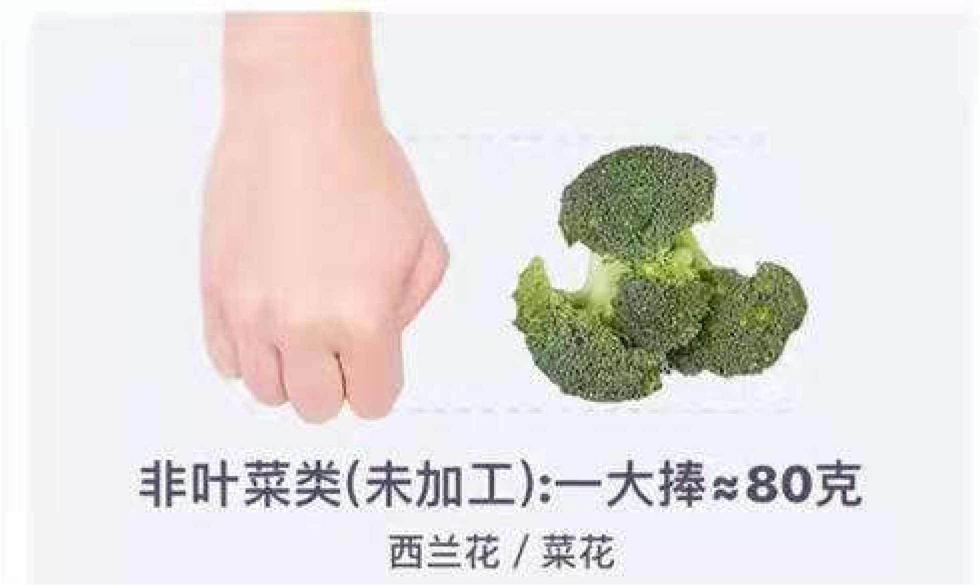

	Basically don't eat	1 time per month	2-3 times per month	1 time per week	2 times a week	3-4 times per week	5-6 times per week	1 time per day	≥2 times per day
Onion	○	○	○	○	○	○	○	○	○
Garlic sprouts	○	○	○	○	○	○	○	○	○
Onion	○	○	○	○	○	○	○	○	○
Chives	○	○	○	○	○	○	○	○	○

13. How often do you eat the following other vegetables? How many grams do you eat each time? (Please estimate based on the picture prompt and answer in grams in the option description.) [Matrix Single Choice] *

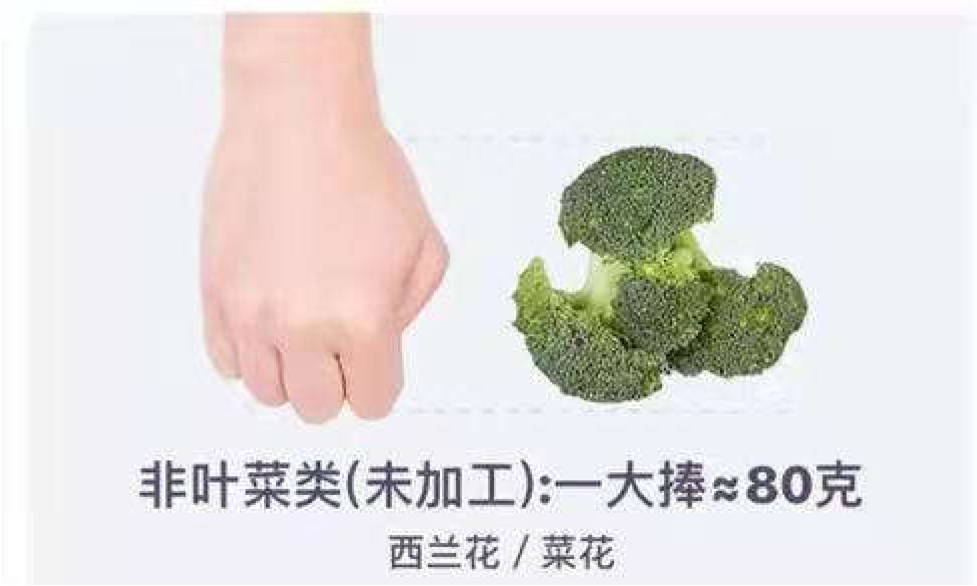

	Basically don't eat	1 time per month	2-3 times per month	1 time per week	2 times a week	3-4 times per week	5-6 times per week	1 time per day	≥2 times per day
Carrots	○	○	○	○	○	○	○	○	○
White Radish	○	○	○	○	○	○	○	○	○
Red Pepper	○	○	○	○	○	○	○	○	○
Green Chili	○	○	○	○	○	○	○	○	○
colored peppers	○	○	○	○	○	○	○	○	○
Tomatoes	○	○	○	○	○	○	○	○	○
Yellow Cauliflower	○	○	○	○	○	○	○	○	○

Eggplant	○	○	○	○	○	○	○	○	○
Yam	○	○	○	○	○	○	○	○	○
root	○	○	○	○	○	○	○	○	○

14. How often do you eat the following mushrooms and algae? How many grams do you eat each time? (Please estimate based on the picture prompt and answer in grams in the option description.) [Matrix single-choice] *

	Basically don't eat	1 time per month	2-3 times per month	1 time per week	2 times a week	3-4 times per week	5-6 times per week	1 time per day	≥2 times per day
Mushroom	○	○	○	○	○	○	○	○	○
Shiitake Mushroom	○	○	○	○	○	○	○	○	○
Tea Tree Mushroom	○	○	○	○	○	○	○	○	○
Apricot mushroom	○	○	○	○	○	○	○	○	○
Black fungus	○	○	○	○	○	○	○	○	○
Skunk cabbage	○	○	○	○	○	○	○	○	○
Nori	○	○	○	○	○	○	○	○	○
Kelp	○	○	○	○	○	○	○	○	○

15. How often do you eat the following fruits? How many grams do you eat each time? (Please estimate based on the picture prompt and answer in grams in the option description.) [Matrix Single Choice] *

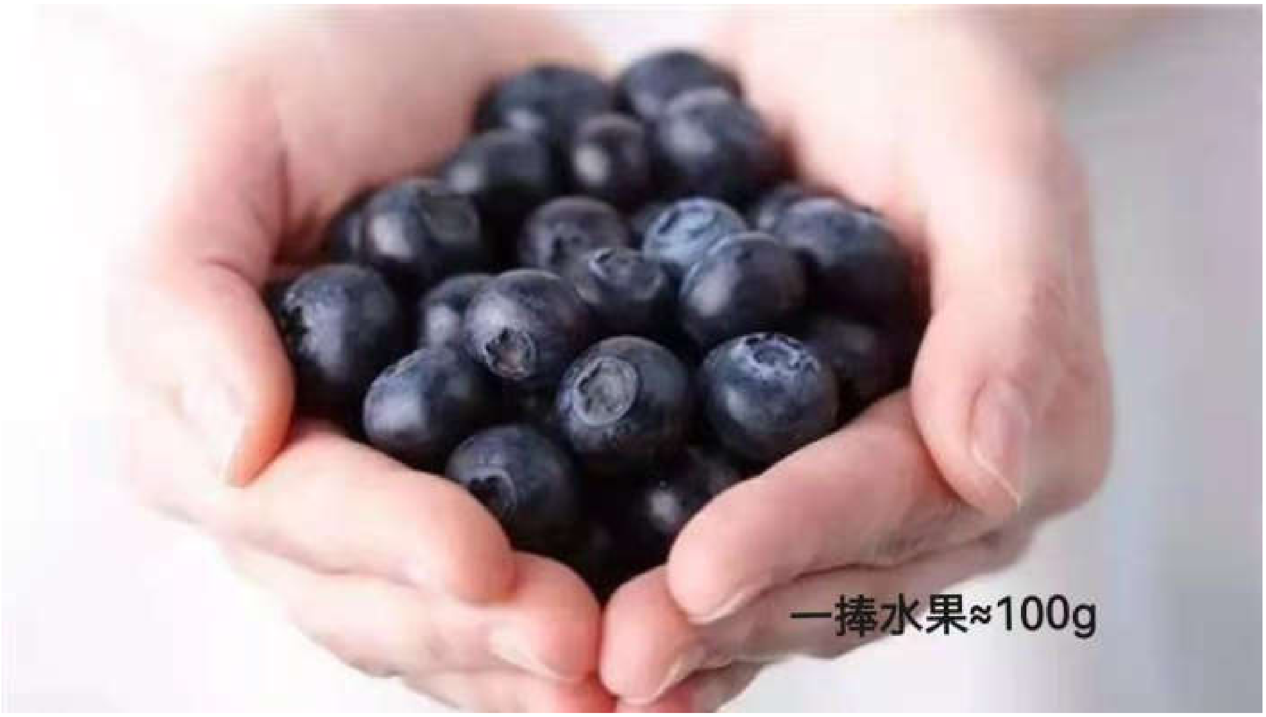

	Basically don't eat	1 time per month	2-3 times per month	1 time per week	2 times a week	3-4 times per week	5-6 times per week	1 time per day	≥2 times per day
Apple	○	○	○	○	○	○	○	○	○
Banana	○	○	○	○	○	○	○	○	○
Strawberry	○	○	○	○	○	○	○	○	○
Persimmon	○	○	○	○	○	○	○	○	○
Red fruit (Hawthorn)	○	○	○	○	○	○	○	○	○
Grapes	○	○	○	○	○	○	○	○	○
Orange	○	○	○	○	○	○	○	○	○
Citrus	○	○	○	○	○	○	○	○	○

Pineapple	○	○	○	○	○	○	○	○	○
Mangosteen	○	○	○	○	○	○	○	○	○
Dragon Fruit	○	○	○	○	○	○	○	○	○
Dates (dried)	○	○	○	○	○	○	○	○	○
Dates (fresh)	○	○	○	○	○	○	○	○	○
Peach	○	○	○	○	○	○	○	○	○
Apricot	○	○	○	○	○	○	○	○	○
Pears	○	○	○	○	○	○	○	○	○

16. How often do you eat the following nuts? How many grams do you eat each time? (Please estimate based on the picture prompt, grams are answered in the option description.) [Matrix Single Choice] *

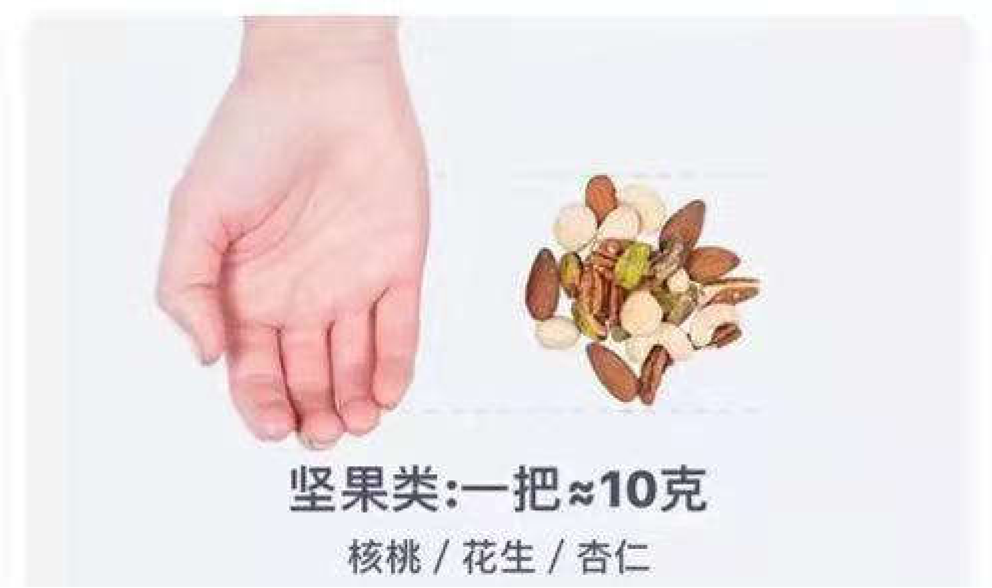

	Basic ally	1 time per	2-3 times	1 time per	2 times	3-4 times	5-6 times	1 time per	≥2 times

	don't eat	month	per month	week	a week	per week	per week	day	per day
Walnuts	○	○	○	○	○	○	○	○	○
Almonds (raw)	○	○	○	○	○	○	○	○	○
Almonds (cooked)	○	○	○	○	○	○	○	○	○
Chestnuts	○	○	○	○	○	○	○	○	○
Cashew nuts	○	○	○	○	○	○	○	○	○
Lotus seeds	○	○	○	○	○	○	○	○	○
Batamax	○	○	○	○	○	○	○	○	○
Pistachio	○	○	○	○	○	○	○	○	○
Hazelnut	○	○	○	○	○	○	○	○	○
Peanuts (nuts)	○	○	○	○	○	○	○	○	○
Sunflower seeds	○	○	○	○	○	○	○	○	○
Pumpkin seeds	○	○	○	○	○	○	○	○	○
Watermelon seeds	○	○	○	○	○	○	○	○	○

17. How often do you eat the following fish, shrimp and crab? How many grams do you eat each time? (Please estimate based on the picture prompt and answer in grams in the option description.) [Matrix Single Choice] *

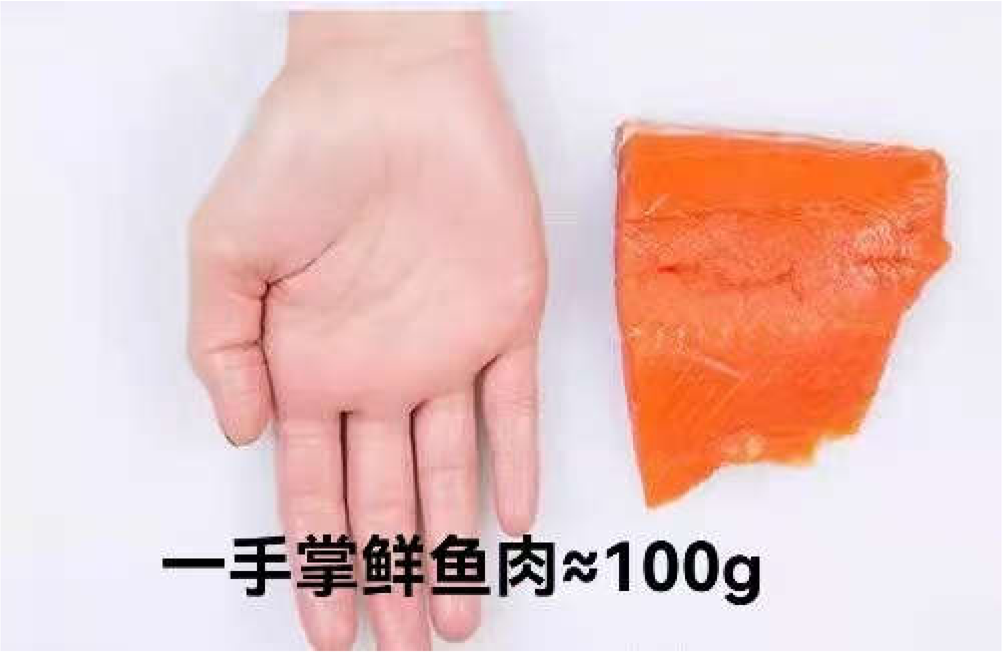

	Basically don't eat	1 time per month	2-3 times per month	1 time per week	2 times a week	3-4 times per week	5-6 times per week	1 time per day	≥2 times per day
Grass carp	○	○	○	○	○	○	○	○	○
Crucian carp	○	○	○	○	○	○	○	○	○
Scallops	○	○	○	○	○	○	○	○	○
Pomfret	○	○	○	○	○	○	○	○	○
Yellowtail	○	○	○	○	○	○	○	○	○
Freshwater shrimp	○	○	○	○	○	○	○	○	○
Sea shrimp	○	○	○	○	○	○	○	○	○
Freshwater Crab	○	○	○	○	○	○	○	○	○

Sea Crab	○	○	○	○	○	○	○	○	○

18. How often do you eat the following animal offal? How many grams do you eat each time? (Please estimate based on the picture prompt and answer in grams in the option description.) [Matrix Single Choice] *

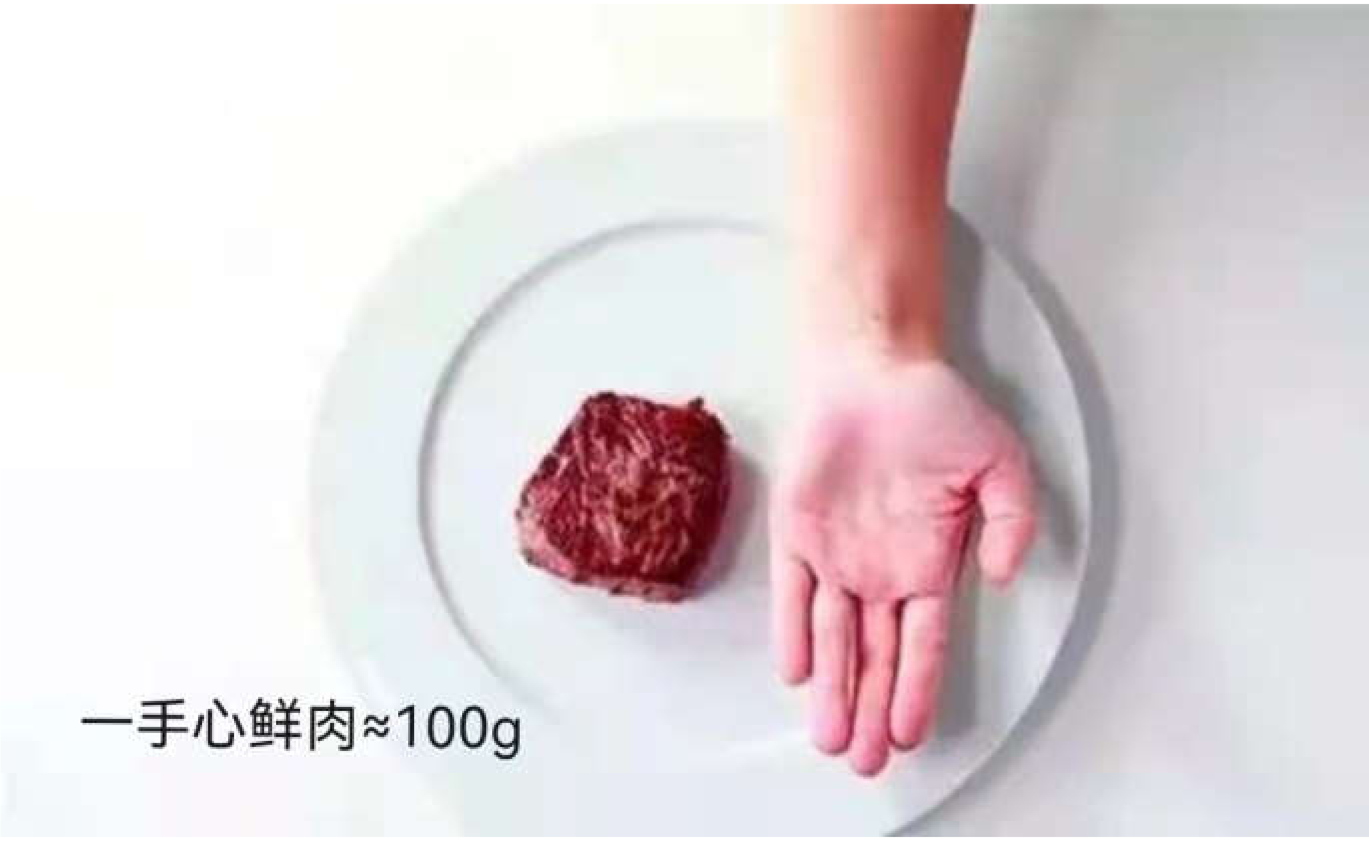

	Basically don't eat	1 time per month	2-3 times per month	1 time per week	2 times a week	3-4 times per week	5-6 times per week	1 time per day	≥2 times per day
Pork Liver	○	○	○	○	○	○	○	○	○
Pork kidney (loin)	○	○	○	○	○	○	○	○	○
beef tripe	○	○	○	○	○	○	○	○	○

Lamb Liver	○	○	○	○	○	○	○	○	○
Chicken Liver	○	○	○	○	○	○	○	○	○
Chicken gizzard/heart	○	○	○	○	○	○	○	○	○

19. How often do you eat the following meat from livestock? How many grams do you eat each time? (Please estimate based on the picture prompt and answer in grams in the option description.) [Matrix Single Choice] *

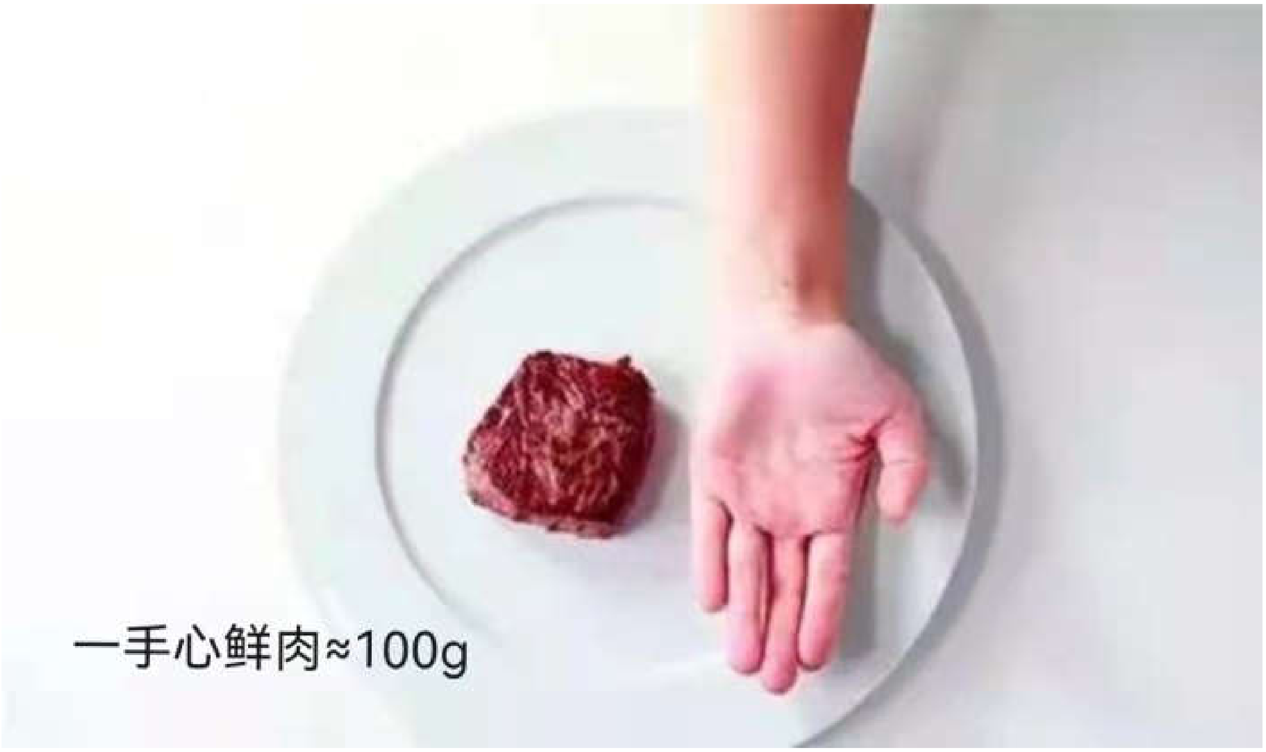

	Basically don't eat	1 time per month	2-3 times per month	1 time per week	2 times a week	3-4 times per week	5-6 times per week	1 time per day	≥2 times per day
Pork	○	○	○	○	○	○	○	○	○

Lamb	○	○	○	○	○	○	○	○	○
Beef	○	○	○	○	○	○	○	○	○
Chicken	○	○	○	○	○	○	○	○	○
Roast Duck	○	○	○	○	○	○	○	○	○
Roasted Squab	○	○	○	○	○	○	○	○	○

20. How often do you eat the following dairy products and their products? How many **grams do you** eat each time? (Milk in 250ml for one cup, yogurt in 100g for one cup, condensed milk and cheese in teaspoon for 20g) [Matrix single-choice] *

	Basically don't eat	1 time per month	2-3 times per month	1 time per week	2 times a week	3-4 times per week	5-6 times per week	1 time per day	≥2 times per day
Milk	○	○	○	○	○	○	○	○	○
Yogurt	○	○	○	○	○	○	○	○	○
Condensed milk	○	○	○	○	○	○	○	○	○
Cheese	○	○	○	○	○	○	○	○	○

21. How often do you eat the following eggs? How **many** do you eat each time? [Matrix Multiple Choice] *

	Basically don't eat	1 time per month	2-3 times per month	1 time per week	2 times a week	3-4 times per week	5-6 times per week	1 time per day	≥2 times per day

Eggs	○	○	○	○	○	○	○	○	○
Salted Duck Egg	○	○	○	○	○	○	○	○	○
preserved egg	○	○	○	○	○	○	○	○	○

22. How often do you eat the following beverages? How many cups do you eat each time? (One cup intake is 250ml) [Matrix single-choice] *

	Basically don't eat	1 time per month	2-3 times per month	1 time per week	2 times a week	3-4 times per week	5-6 times per week	1 time per day	≥2 times per day
Sugary drinks	○	○	○	○	○	○	○	○	○
Ice Cream	○	○	○	○	○	○	○	○	○
Tea	○	○	○	○	○	○	○	○	○
Coffee	○	○	○	○	○	○	○	○	○

23. How often do you eat fried foods? (Fruits, rolled rings, fried cakes, fried eggplant clips/root clips, fried nuts, fried fava beans, fried balls, fried chicken legs) [Matrix multiple choice] *

	Basically don't eat	1 time per month	2-3 times per month	1 time per week	2 times a week	3-4 times per week	5-6 times per week	1 time per day	≥2 times per day
Fried food	○	○	○	○	○	○	○	○	○

24. How often do you eat grilled food? (Lamb kebabs, grilled meat) [Matrix single-choice] *

	Basically don't eat	1 time per month	2-3 times per month	1 time per week	2 times a week	3-4 times per week	5-6 times per week	1 time per day	≥2 times per day
Barbecue food	○	○	○	○	○	○	○	○	○

25. How often do you eat smoked foods? (smoked sausage, salami/bacon) [Matrix single-choice] *

	Basically don't eat	1 time per month	2-3 times per month	1 time per week	2 times a week	3-4 times per week	5-6 times per week	1 time per day	≥2 times per day
Smoked foods	○	○	○	○	○	○	○	○	○

26. Do you take any nutritional supplements regularly? How often do you take them? On average, how much do you take each time? [Matrix Multiple Choice] *

	Basically don't eat	1 time per month	2-3 times per month	1 time per week	2 times a week	3-4 times per week	5-6 times per week	1 time per day	≥2 times per day
Multivitamin tablets	○	○	○	○	○	○	○	○	○
B vitamin tablets	○	○	○	○	○	○	○	○	○
E Vitamin	○	○	○	○	○	○	○	○	○

Tablets									
C vitamin tablets	○	○	○	○	○	○	○	○	○
D vitamin tablets	○	○	○	○	○	○	○	○	○
Micronutrient supplements (45 questions to fill in the name)	○	○	○	○	○	○	○	○	○
Others: ____________	○	○	○	○	○	○	○	○	○

*Finish the question and skip to question **27.**

27. names of other types of nutritional supplements [fill in the blank]*

____

28. Which two cooking methods do you eat most often? [Multiple choice] *

□ Frying

□ Red Hot

□ Frying

□ Steamed

□ pickled

□Cooking and stewing

□ Baking

□ Other: ____________ _________________
