# Supplementary material for: Dietary Fiber Intake Influences Changes in Ankylosing Spondylitis Disease Status
Source: J Clin Med. 2023 Feb 17;12(4):1621. doi: 10.3390/jcm12041621 (PMC9960917; doi:10.3390/jcm12041621)
Supplement: Supplementary file 1 [file jcm-12-01621-s001.zip › Supplementary Table1.pdf]

SupplementaryTable S1: Univariable associations between dietary fiber and assessments of AS

	Univariate analysis			
	Low n=93 (56.36%)	High n=72 (43.64%)		
		β	95%CI	P-value
ESR	ref	-10.9	(-16.6, -5.1)	< 0.01
CRP	ref	-8.8	(-12.4, -5.2)	< 0.01
ASDAS-CRP	ref	-1.0	(-1.2, -0.7)	< 0.01
BASDAI	ref	-1.0	(-1.2, -0.7)	< 0.01
ASAS-HI	ref	-1.8	(-2.8, -0.7)	< 0.01
BASMI	ref	0.1	(-0.4, 0.6)	0.73
BASFI	ref	-0.8	(-1.1, -0.4)	< 0.01

Missing data, n (%): CRP 8 (5.2%), ESR 10 (6.5%), ASDAS-CRP 8 (5.2%), BASMI 10 (6.5%). BMI: body mass index; HLA-B27: human leucocyte antigen B-27; CRP: C-reactive protein; ESR: erythrocyte sedimentation rate; ASDAS-CRP: Ankylosing Spondylitis Disease Activity Score based on CRP; BASDAI: Bath Ankylosing Spondylitis Disease Activity Index; ASAS HI: Assessment of Spondyloarthritis International Society Health Index; BASFI: Bath Ankylosing Spondylitis Functional Index; BASMI: Bath Ankylosing Spondylitis Metrology Index
