# Supplementary material for: Dietary Fiber Intake Influences Changes in Ankylosing Spondylitis Disease Status
Source: J Clin Med. 2023 Feb 17;12(4):1621. doi: 10.3390/jcm12041621 (PMC9960917; doi:10.3390/jcm12041621)
Supplement: Supplementary file 1 [file jcm-12-01621-s001.zip › Supplementary Table2.pdf]

Supplementary Table S2 Relationship between Dietary fiber intake and BASMI

	Dietary fiber intake			
**BASMI**	Low	High		
**Crude Model**		β	95%CI	P-value
FBD symptoms (+)	ref	0.2	(-0.5, 0.9)	0.60
FBD symptoms (-)	ref	0.2	(-0.6, 0.9)	0.70
Total	ref	0.2	(-0.4, 0.7)	0.55
**Model I**				
FBD symptoms (+)	ref	0.5	(-0.3, 1.4)	0.24
FBD symptoms (-)	ref	0.5	(-0.3, 1.3)	0.27
Total	ref	0.5	(-0.1, 1.1)	0.08
**Model II**				
FBD symptoms (+)	ref	0.5	(-0.3, 1.4)	0.24
FBD symptoms (-)	ref	0.5	(-0.3, 1.3)	0.27
Total	ref	0.5	(-0.1, 1.1)	0.08

Model I adjusted for: sex; age; Nsaids-use; TNFi-use; BMI; lnESR; lnCRP; PsA
Model **II** adjusted for: sex; age Nsaids-use; TNFi-use; BMI; lnESR; lnCRP; PsA
Missing data, n (%): CRP 8 (5.2%), ESR 10 (6.5%), FBD 12 (7.3%). BASMI 10 (6.5%) BMI: body mass index; CRP: C-reactive protein; ESR: erythrocyte sedimentation rate; BASMI: Bath Ankylosing Spondylitis Metrology Index; FBD: functional bowel disorder; NSAID: non-steroidal anti-inflammatory drug; TNFi: tumor necrosis factor inhibitor; PsA: psoriatic arthritis
