# Supplementary material for: Dietary Fiber Intake Influences Changes in Ankylosing Spondylitis Disease Status
Source: J Clin Med. 2023 Feb 17;12(4):1621. doi: 10.3390/jcm12041621 (PMC9960917; doi:10.3390/jcm12041621)
Supplement: Supplementary file 1 [file jcm-12-01621-s001.zip › Supplementary Table3.pdf]

**Supplementary Table S3** Relationship between Dietary fiber intake and BASFI

| | Dietary fiber intake | | | |
|---|---|---|---|---|
| **BASFI** | Low | High | | |
| **Crude Model** | | β | 95%CI | P-value |
| FBD symptoms（+） | ref | -1.0 | (-1.7, -0.3) | < 0.01 |
| FBD symptoms（-） | ref | -0.5 | (-1.0, 0.0) | 0.08 |
| Total | ref | -0.7 | (-1.1, -0.3) | < 0.01 |
| **Model I** | | | | |
| FBD symptoms（+） | ref | -0.5 | (-1.2, 0.3) | 0.24 |
| FBD symptoms（-） | ref | -0.4 | (-1.0, 0.2) | 0.16 |
| Total | ref | -0.4 | (-0.9, 0.0) | 0.07 |
| **Model II** | | | | |
| FBD symptoms（+） | ref | -1.0 | (-1.7, -0.3) | < 0.01 |
| FBD symptoms（-） | ref | -0.2 | (-0.7, 0.4) | 0.56 |
| Total | ref | -0.4 | (-0.9, 0.1) | 0.10 |

Model I adjusted for: sex; age; lnESR; lnCRP

Model **II** adjusted for: sex; age; CM -use; TNFi-use; BMI; lnESR; lnCRP

Missing data, n (%): ASDAS-CRP 8 (5.2%), CRP 8 (5.2%), ESR 10 (6.5%), FBD 12 (7.3%). BMI: body mass index; CRP: C-reactive protein; ESR: erythrocyte sedimentation rate; BASFI: Bath Ankylosing Spondylitis Functional Index; FBD: functional bowel disorder; TNFi: tumor necrosis factor inhibitor; CM: Chinese medicine
