# Supplementary material for: Dietary Fiber Intake Influences Changes in Ankylosing Spondylitis Disease Status
Source: J Clin Med. 2023 Feb 17;12(4):1621. doi: 10.3390/jcm12041621 (PMC9960917; doi:10.3390/jcm12041621)
Supplement: Supplementary file 1 [file jcm-12-01621-s001.zip › Supplementary Table4.pdf]

Supplementary Table S4 Relationship between Dietary fiber intake and ASAS-HI

	Dietary fiber intake			
**ASAS-HI**	Low	High		
**Crude Model**		β	95%CI	P-value
FBD symptoms（+）	ref	-2.6	(-4.2, -1.0)	<0.01
FBD symptoms（-）	ref	-0.7	(-2.0, 0.7)	0.31
Total	ref	-1.4	(-2.5, -0.4)	<0.01
**Model I**				
FBD symptoms（+）	ref	-1.9	(-3.6, -0.2)	0.03
FBD symptoms（-）	ref	-1.0	(-2.6, 0.6)	0.23
Total	ref	-1.4	(-2.6, -0.2)	0.02
**Model II**				
FBD symptoms（+）	ref	-2.1	(-4.0, -0.1)	0.04
FBD symptoms（-）	ref	-0.6	(-2.1, 0.9)	0.44
Total	ref	-1.2	(-2.4, 0.0)	0.05

Model I adjusted for: sex; age; lnCRP; BMI

Model **II** adjusted for: sex; age; CM-use; NSAID-use; BMI; lnESR; lnCRP

Missing data, n (%): CRP 8 (5.2%), ESR 10 (6.5%), FBD 12 (7.3%). BMI: body mass index; CRP: C-reactive protein; ESR: erythrocyte sedimentation rate; ASAS HI: Assessment of Spondyloarthritis International Society Health Index; FBD: functional bowel disorder; NSAID: nonsteroidal anti-inflammatory drug; CM: Chinese medicine
